# Modern Radiation Therapy for the Management of Brain Metastases From Non-Small Cell Lung Cancer: Current Approaches and Future Directions

**DOI:** 10.3389/fonc.2021.772789

**Published:** 2021-11-02

**Authors:** Cristina Mantovani, Alessio Gastino, Marzia Cerrato, Serena Badellino, Umberto Ricardi, Mario Levis

**Affiliations:** Department of Oncology, University of Torino, Torino, Italy

**Keywords:** brain metastases, radiosurgery, SRS, WBRT (whole brain radiotherapy), modern radiotherapy, leptomeningeal dissemination, NSCLC, hippocampal avoidance

## Abstract

Brain metastases (BMs) represent the most frequent event during the course of Non-Small Cell Lung Cancer (NSCLC) disease. Recent advancements in the diagnostic and therapeutic procedures result in increased incidence and earlier diagnosis of BMs, with an emerging need to optimize the prognosis of these patients through the adoption of tailored treatment solutions. Nowadays a personalized and multidisciplinary approach should rely on several clinical and molecular factors like patient’s performance status, extent and location of brain involvement, extracranial disease control and the presence of any “druggable” molecular target. Radiation therapy (RT), in all its focal (radiosurgery and fractionated stereotactic radiotherapy) or extended (whole brain radiotherapy) declinations, is a cornerstone of BMs management, either alone or combined with surgery and systemic therapies. Our review aims to provide an overview of the many modern RT solutions available for the treatment of BMs from NSCLC in the different clinical scenarios (single lesion, oligo and poly-metastasis, leptomeningeal carcinomatosis). This includes a detailed review of the current standard of care in each setting, with a presentation of the literature data and of the possible technical solutions to offer a “state-of-art” treatment to these patients. In addition to the validated treatment options, we will also discuss the future perspectives on emerging RT technical strategies (e.g., hippocampal avoidance whole brain RT, simultaneous integrated boost, radiosurgery for multiple lesions), and present the innovative and promising findings regarding the combination of novel targeted agents such as tyrosine kinase inhibitors and immune checkpoint inhibitors with brain irradiation.

## Introduction

Brain metastases (BMs) represents the most frequent Central Nervous System (CNS) neoplasm and Non-Small Cell Lung Cancer (NSCLC) accounts for approximately 50% of the lesions.

In NSCLC, 10-25% of patients present BMs at the time of diagnosis, and up to 50% develop them during the disease course, with increasing incidence in recent years owing to advances in diagnostic and therapeutic procedures (1, 2).

Globally, the prognosis in this setting remains severe (inferior to 3 months without any treatments), and the prognostic stratification of these patients is crucial for an optimal management (3).

In the last 20 years we observed the creation and evolution of different prognostic scoring systems, aiming to guide clinicians to offer tailored treatments (4–7).

Starting from the Recursive Partitioning Analysis (RPA), based on age, Karnosfky Performance Status (KPS), control of the primary tumor and presence of extracranial metastases, in 2008 Sperduto et al. proposed the Graded Prognostic Assessment (GPA) which considers also the number of BMs (4, 5). Finally, the adoption of NSCLC-specific prognostic indices, integrated with molecular data, defined the modern (GPA) for Lung Cancer Using Molecular Markers (Lung-molGPA) (6, 7).

In the era of precision medicine, radiation therapy (RT) still represents a cornerstone of BMs management, and the two major radiotherapeutic options, whole brain radiotherapy (WBRT) and focal radiotherapy, are perfectly integrated with surgery and systemic therapies in a multimodal approach (8). Given the detrimental effect of BMs on the prognosis of NSCLC patients, the selection of the appropriate treatment on a case-by-case judgment is of pivotal importance to obtain the remission of pre-existing BMs, to prevent the development of new lesions and to possibly improve the final outcome.

Our review provides an overview of the actual indications to RT treatments in the management of BMs from NSCLC in the different clinical scenarios (single lesion, oligometastatic disease, polymetastatic disease, leptomeningeal carcinomatosis and prophylactic cranial irradiation (PCI) in locally advanced disease), with a secondary focus on future perspectives on emerging technical RT solutions and their possible combinations with novel targeted agents.

## Single Lesion

Single BM represents the most favorable disease presentation in the setting of BMs.

Several randomized controlled trials (RCTs) demonstrated that the radical management of the single lesion with focal treatments, either surgery or RT, in addition to the historical WBRT approach, improves both local control (LC) and overall survival (OS) (9–11).

Surgery is the gold standard treatment for large, edemigenous lesions, allowing an immediate symptoms relief and, when necessary, a histological determination (12). In resected patients, RT is universally adopted as adjuvant therapy to reduce the risk of local relapse.

Radiosurgery (SRS), a highly conformal technique delivering high doses of radiation in a single fraction, represents the best solution for small lesions and a valid option for larger lesions when surgery is not feasible (generally for comorbidities or involvement of brain areas with high risk of post-surgical sequelae).

SRS can be delivered with different technical solutions such as Gamma-knife, Cyber-knife, LINAC-based SRS and none of these have been shown to be superior (13).

## Upfront Focal RT

The potential of SRS as an alternative focal strategy to surgery was initially described with case reports between the 1980s and 1990s (14, 15).

The first robust evidence came from the RTOG 9508 trial. In this multi-institutional study, 333 patients with 1 to 3 BMs were randomized to receive WBRT or WBRT + SRS boost; 186 patients (56% of the total) had single brain lesion. In the overall population, the addition of SRS achieved an advantage in LC (1-y LC: 81% WBRT+SRS *vs* 71% WBRT alone, p=0.01) without any survival benefit. After stratification for the number of brain lesions, patients with a single BM had a superior median OS with the addition of SRS (6.5 vs 4.9 months, p=0.039) (11).

The next generation of RCTs strengthened the role of upfront SRS, proposing the omission of WBRT in patients with oligometastatic disease (1-4 lesions) (16–19). Approximately 50% of the enrolled population of these studies had only a single lesion (range 48%-67%). The results, despite the different primary endpoints considered, were univocal and consistent with the surgical series (20, 21), showing better intracranial disease control in patients who received additional whole brain irradiation, but without a translation into a survival advantage. At the same time, WBRT negatively influenced some aspects of the quality of life (QoL) and the neurocognitive functions of these patients (16, 17, 19, 22). All the historical RCTs considered so far proposed a single-high-dose fraction for lesions with a maximum diameter of less than 3-4 cm (11, 16–19).

The maximum tolerated single fraction dose was established with a risk adapted approach in the RTOG 9005 phase I dose escalation trial. Maximum tolerated doses were 24 Gy, 18 Gy, and 15 Gy for tumors < 20 mm, 21–30 mm, and 31–40 mm in maximum diameter, respectively (23, 24).

This risk adaptive approach is currently adopted in clinical practice, although for larger lesions (>2 cm) 18-15 Gy or less in single fraction could be detrimental in terms of LC. On the other hand, greater RT doses could lead to an excessive risk of complications, in particular radionecrosis (RN).

**Figure 1:**
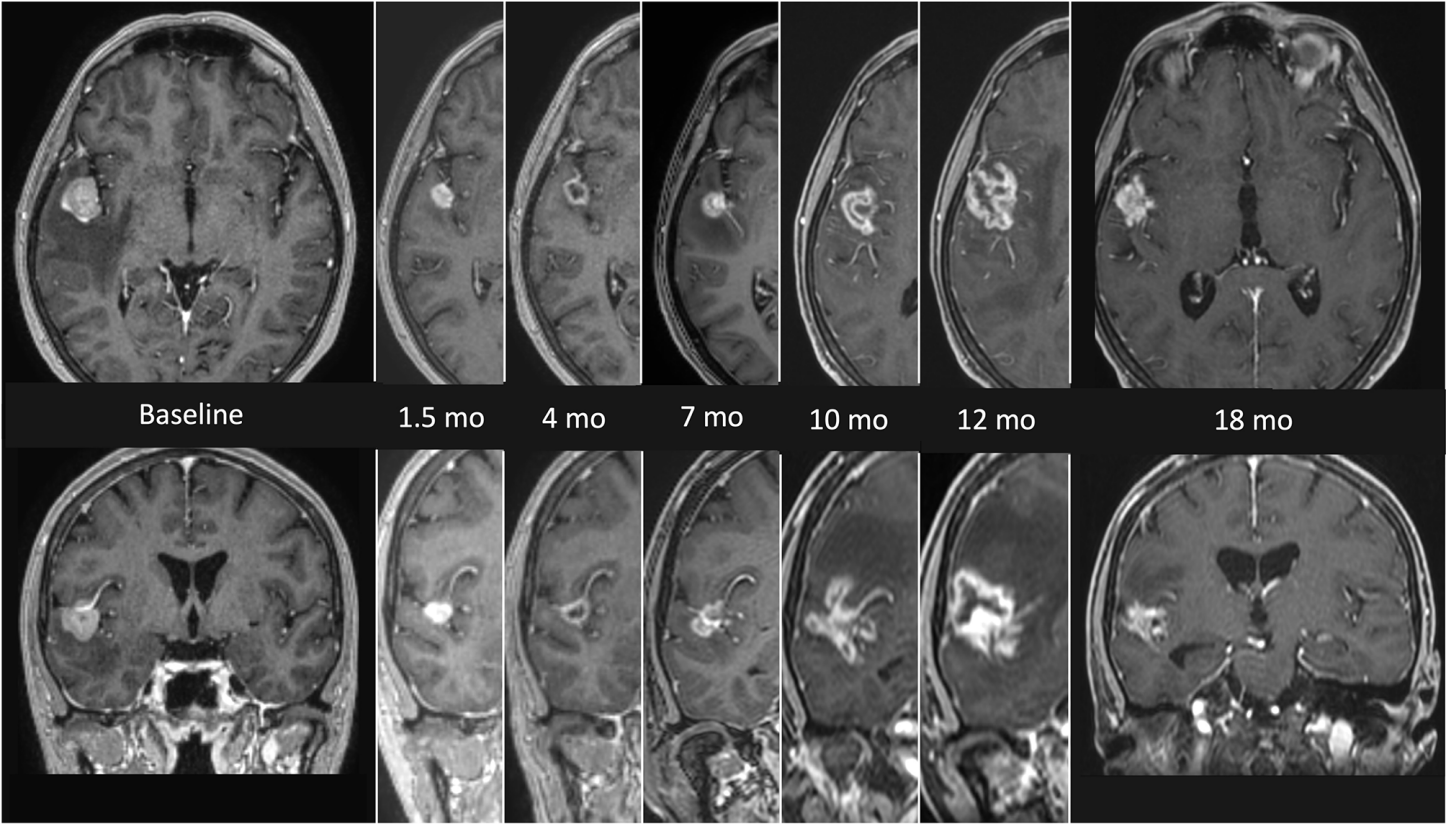
MRI findings presenting the evolution of radionecrosis (RN) within a timeframe of 18 months from the end of radiosurgery (SRS) in a single right temporal BM from NSCLC. The first images on the left (axial and coronal) represent the lesion before focal treatment. Serial follow up images show an initial shrinkage of the treated lesion followed by a significant and continuous increase of the necrotic component. The latest images on the right (axial and coronal) refers to the situation 18 months after SRS, with a clear reduction of the necrotic component, which allows a differential diagnosis with a relapse.

RN represents the major complication of SRS, with a highly variable incidence rate in the literature (range 5-25%) (25). The time to occurrence varies from few months to several years after radiation, but approximately 80% of cases occur within 3 years (26). The etiology is multifactorial, mainly related to the exposure of a significant volume of healthy brain tissue to high doses of radiation, previous brain irradiation, concomitant systemic therapies and some specific histologies (27). The radiological aspect is characterized by enhancing lesions and/or rounded presentations with intra-lesional areas of necrosis in the previously irradiated brain tissue. Standard neuroimaging may be inadequate to distinguish RN from tumor progression, requiring more advanced Magnetic Resonance Imaging (MRI) sequences and functional imaging (25). **Figure 1** shows a typical sequence of MR findings of RN occurred after SRS. The therapeutic approach, particularly relevant in highly symptomatic presentations, ranges from the classic steroid therapy to the use of bevacizumab, up to the need for surgical resection for large, edemigenous lesions (28).

The ideal solution to improve LC without increasing the risk of RN related to focal RT is represented by (hypo-)fractionated stereotactic RT (SRT). By delivering a lower dose per fraction in few (generally ≤ 5) sessions, it is possible to achieve higher biologically effective doses (BED) to the tumor with a lower radiobiological effect to the surrounding brain tissue. This approach became possible as a result of the introduction of Image-Guided RT (IGRT), which enables to deliver high doses of radiation without stereotactic invasive headframe (frameless SRT). Retrospective series of large brain lesions treated with SRT accumulated over the years, providing better rates of LC (1-y LC: 79.2%-92.9%) and RN (RN rates: 6.5%-7.3%) compared with SRS (29).

Data on Tumor Control Probability (TCP), considered as the probability of LC (30) and Normal Tissue Complication Probability (NTCP), considered as the risk of RN (31), were recently published as Organ-Specific Papers from the international collaborative project “HYTEC” (Hypofractionated Treatment Effects in the Clinic), to guide dose and fractionation choices for SRS/SRT in BMs. The authors concluded that single-fraction SRS with doses of 18-24 Gy should be the first choice for tumors ≤ 2 cm, guaranteeing an estimated LC of 85%-95% at 12 months, while SRT should be preferred for lesion >2 cm (30). The risk of RN is modeled on dose/volume parameters. For SRS, the volume of healthy brain tissue receiving 12 Gy (V12) is a strong predictor of symptomatic RN, with a risk lowered to 10% when less than 5 cm^3^ are exposed to that threshold dose. For SRT, the volume of healthy tissues that might be exposed to high radiation doses is even larger, thus representing the best schedule for bigger lesions (31). Ongoing trials, reported in **Table 1**, will provide further evidences. The choice of the focal strategy between surgery and SRS in resectable lesions deserves further considerations. To date, there is no evidence from randomized trials directly comparing the two treatment modalities.

**Table 1:** Currently ongoing clinical trials investigating RT for oligometastatic disease.

NCT identifier		Study phase	Patients estimated		Study population	Standard arm	Experimental arm	Primary endpoint
***Upfront focal RT***								
NCT04805255 (Chang Gung Memorial Hospital)	NA (Cohort study)			135	1-3 BMs ≤4.0 cm diameter	–	SRT (30-35 Gy/5 fx)	Cognitive-deterioration-free survival
NCT02054689 (University of Pittsburgh)	I			25	Large BM (3-5 cm), maximum 3 lesions	–	SRT (24 to 36 Gy in 3 fx, 8-12 Gy/fx)	MTD
NCT03726359 (Albert Einstein College of Medicine)	I			43	Large BM (3-5 cm)	–	SRT (starting dose 35 Gy/5 fx every other day)	MTD
NCT03412812 (University of Alabama at Birmingham)	I			60	Large BMs (2.1-6 cm)	–	SRT (5 fx)	MTD
NCT02747303 (University of Chicago)	II			166	1-5 BMs ≤3.0 cm diameter	SRS	0 mm GTV-PTV margins SRS	PFS
NCT03697343 (University of Erlangen-Nürnberg Medical School)	III			302 (not yet recruiting)	Large BMs (2-4 cm) maximum 4 lesions	SRS	SRT	Time to local progression
***Adjuvant SRS/SRT***								
NCT03285932 (ESTRON)		II	50		Resected BM and ≤10 unresected lesions	WBRT	SRT (35 Gy/7fx) + SRS/SRT for any unresected lesions	Neurological PFS
NCT03561896 (Oncology Institute of Southern Switzerland)		II	60		Resected BM	SRS	IGRT (SRT)	Relapse rate
NCT04114981 (Alliance)		III	208		Resected BMs	SRS	SRT	Surgical bed recurrence-free survival
***Neoadjuvant SRS***								
NCT03163368 (Cedars-Sinai Medical Center)		I	25		Resectable BMs	–	Neoadjuvant SRS dose escalation	MTD
NCT01891318 (Case Comprehensive Cancer Center)		I-II	36		Resectable BMs	–	Neoadjuvant SRS	MTD, LC
NCT04503772 (Centre Jean Perrin)		II	70		Resectable BMs	–	Neoadjuvant SRT (33 Gy/3 fx at the isocenter)	6mo-LC
NCT04422639 (University of Arkansas)		II	104		Resectable BMs	Adjuvant SRS/SRT	Neoadjuvant SRS/SRT	Time to CNS Composite Event (LR, symptomatic RN, LMD)
NCT03741673 (MDACC)		III	86		Resectable BMs	Adjuvant SRS	Neoadjuvant SRS	LMD free rate
NCT03750227 (Mayo Clinic)		III	140		Resectable BMs	Adjuvant SRS	Neoadjuvant SRS	CNS composite endpoint event
NCT04474925 (AHS Cancer Control Alberta)		III	88 (not yet recruiting)		Resectable BMs	Adjuvant SRS	Neoadjuvant SRS	LC

NA, not applicable; fx, fractions; MTD, Maximum Tolerated Dose; PFS, Progression Free Survival; LR, Local Relapse; LMD, LeptoMeningeal Dissemination; RN, Radionecrosis; CNS, Central Nervous System.

Two attempts of RCT between SRS and surgery were stopped early for slow accrual: Muacevic et al. randomized 64 patients with a single, small sized (≤3 cm) resectable BM to receive surgery + WBRT vs SRS alone (32); Roos et al. randomized 21 patients with a solitary BM to SRS vs surgery, both with adjuvant WBRT (33).

A Cochrane review of 2018 tried to pool the 85 patients from these two studies, but a meta-analysis was not possible due to clinical heterogeneity between trial interventions (34). More recent studies compared the efficacy of SRS and surgery as single treatment modalities, as per modern trend to omit WBRT in patients with a limited number of BMs. Quigley et al. compared 162 consecutive patients (46% single lesions) that received surgery + SRS boost (49 patients) vs SRS alone (113 patients). Surgery + SRS boost resulted in greater local control and survival when complete resection was achieved (35).

Recently a secondary analysis of the EORTC 22952-26001 trial investigated for any difference in terms of LC among brain oligometastatic patients treated with SRS (154 patients) or surgery (114 patients). After adjustments for site, size, number of lesions, neurological status and extracranial disease, a similar LC resulted between the two groups. Surgically treated patients experienced a higher rate of early (0-3 months) local recurrence, but the relative benefit of SRS decreased with time (36).

**Conclusions:** SRS represents the best focal approach for small or unresectable single brain lesions. Surgery or SRT should be preferred when treating lesions with a larger diameter >2 cm. The adoption of SRT provides a better LC with a low risk of RN when treating larger lesions.

## RT Complementary to Surgery

### Adjuvant WBRT

Adjuvant WBRT has been considered the standard of care after surgery for many decades, with the rationale of improving both local surgical cavity and distant intracranial control. More recently, the advent of highly sensitive neuroimaging, such as multi-parametric brain MRI, made it reasonable to omit adjuvant WBRT, and its related early (hair loss and fatigue) and late (neurocognitive deterioration) complications, when a complete resection is achieved.

Some multicenter RCTs were conducted to investigate the role of WBRT in this setting.

The first one by Patchell and colleagues (20) enrolled 95 patients (60% with a primary diagnosis of NSCLC), receiving a complete-surgical resection of a single BM. WBRT resulted in better intracranial tumor control compared to observation (anywhere recurrence: 18% vs 70%, p<0.001). Furthermore, WBRT determined less neurological deaths (14% adjuvant WBRT vs 44% surgery alone, p=0.03) but no significant differences in functional independence and OS (approximately 10 months in both arms) were observed.

The EORTC 22952-26001 study investigated the role of adjuvant WBRT after surgery or SRS for 1-3 BMs, randomizing patients to WBRT or observation: 359 patients (NSCLC was the primary tumor in 53%) with a limited number of BMs (1–3) were randomized to receive WBRT or to close observation after local therapy, either surgery or SRS. In the surgery group (160 patients) almost all the patients had a single (96%), large (median size 30 mm) metastasis. For these patients, postoperative WBRT significantly reduced the probability of local relapse by 32% (from 59% to 27%, p <0.001) and the probability of distant intracranial relapse by 19% (from 42% to 23%, p=0.001). Again, no difference was found in functional independence and OS (19).

More recently, the Japanese JCOG0504 non-inferiority trial randomly assigned to WBRT or observation and salvage SRS 271 patients (47% diagnosed with NSCLC) with 4 or fewer surgically resected BMs (73% single lesion).

Observation with salvage SRS was not inferior to WBRT in terms of OS (median OS: 15.6 months in both arms, p=0.027), although median intracranial Progression Free Survival (PFS) was shorter (10.4 months for WBRT vs 4 months for salvage SRS). In addition, a deferred brain irradiation with a highly conformal technique resulted in more than half reduction of severe cognitive deterioration for salvage SRS patients (G2-G4 cognitive dysfunction: 16.4% WBRT vs 7.7% salvage SRS) (21).

**Conclusions:** WBRT is usually avoided as adjuvant treatment, considering the absence of survival benefit and the severe impact on neurocognitive functions. Anyway, it remains the best strategy to prevent the onset of new BMs.

### Adjuvant SRS/SRT

Given the suboptimal LC of surgery alone, with an unacceptable high risk of local recurrence (around 50%) (19, 20), and the inescapable severe neurocognitive sequelae of whole brain irradiation, the modern trend is to offer focal postoperative RT to significantly improve the remission rate of the surgical cavity. In the last two decades, several retrospective studies investigated the role of adjuvant SRS to the surgical cavity and a recent meta-analysis, including 3458 patients from 50 studies, showed high LC (1-y LC: 83.7%) and low toxicity profile (RN rate: 6.9%) (37).

Two randomized trials confirmed the efficacy and safety of this treatment. In the study by Mahajan and colleagues (38), the 12-months freedom from local recurrence was 43% in the observation group and 72% in the SRS group (p=0.015), but with no difference in OS. In the NCCTG N107C/CEC.3 multicenter trial 194 patients (59% NSCLC) were randomly assigned to adjuvant SRS or WBRT, with the latter one providing a better time to intracranial tumor progression (median time: 27.5 months vs 6.4 months, p<0.0001), considering both local and distant recurrence. On the other hand, neurocognitive preservation was significantly higher in the SRS group (median cognitive-deterioration-free survival: 3.7 months vs 3 months, p<0.0001) with significant difference in terms of immediate and delayed memory, processing speed and executive function (39). As predictable, OS was comparable between the two arms (12.2 months for SRS vs 11.6 months for WBRT, p=0.70).

Further evidence from the ESTRON German trial (NCT03285932), presenting the same interventional arms, are awaited to provide more robust evidence on the role of SRS in the adjuvant setting after the resection of BMs (40). Although the availability of high quality data in support of the clinical role of adjuvant SRS to the surgical cavity, some practical and technical challenges remain to be solved. First, the optimal dose and fractionation to obtain the ideal balance between LC and risk of RN. Considering that surgery is generally performed for large, symptomatic lesions, it is very common for the treating radiation oncologist to face with large post-operative volumes to be irradiated. The considerations for cavities greater than 2-3 cm in diameter are the same as for large unresected BMs, with SRT representing the best solution. To date, several retrospective and meta-analysis data are available and confirm an excellent risk-benefit balance of focal SRT to the resection cavity (41–45), with a trend for better LC and lower rates of RN compared to SRS (29, 37). The prescribed dose of SRT usually ranges 24-30 Gy in 3-5 fractions in the published series (41, 46). Based on the available data, the HYTEC TCP and NTCP papers provide useful indications on doses and constraints to orientate in the clinical practice, as previously done for the radical and upfront setting (30, 31). The validation of these results in a randomized setting are awaited from the ongoing Alliance trial (NCT04114981), which is randomizing ≥2 cm-sized-operated brain lesions with post-surgical cavities smaller than 5 cm to be irradiated with single fraction SRS or 3-5 fractions SRT (**Table 1**).

The second issue is related to the uncertainties in the target delineation. The dynamic adaptation of the surgical cavity and of the surrounding tissues after surgical resection, cause significant changes in the shape of the target area, with an average cavity/volume reduction after surgery estimated in a range of 15-43% (47). This volume shrinkage and the timing needed to obtain a reasonable post-surgical clinical recovery, influence the timing for the administrations of adjuvant RT, generally performed within maximum 4-6 weeks after surgery.

In 2017 a consensus contouring guideline for adjuvant SRS in completely resected BM cavity was generated, with the aim of standardizing this highly variable procedure. This paper recommended to use pre- and post-operative contrast-enhancing T1-weighted MRI to guide the contouring process and to include in the clinical target volume (CTV) the entire surgical cavity as well as the surgical tract, with the inclusion of any site of preoperative dural or venous sinus involvement (48).

The last important consideration on post-surgical SRS/SRT is about the risk of leptomeningeal dissemination (LMD). Surgical procedures may disseminate cells in surrounding tissues causing the neoplastic seeding of the cerebrospinal fluid, and focal adjuvant RT seems to be less effective than whole-brain irradiation to prevent this complication. In retrospective series LMD incidence ranges from 8 to 13% (49, 50). In the “Leptomeningeal Metastasis” section this scenario will be further explored.

**Conclusions:** focal RT is currently the preferred adjuvant treatment after surgical resection of BM. The excellent LC and a good toxicity profile make this strategy preferable to WBRT. The ideal timing, doses, fractionations and volumes for post-operative stereotactic irradiation are currently under investigation.

### Neoadjuvant SRS

The new frontier in the treatment of single or few BMs is preoperative SRS. The rational is to sterilize any microscopic disease before the macroscopic resection of a brain lesion and to prevent all critical issues related to adjuvant SRS. Generally, neoadjuvant RT is performed as a single fraction of 16 Gy, given a few days before surgery.

**Figure 2:**
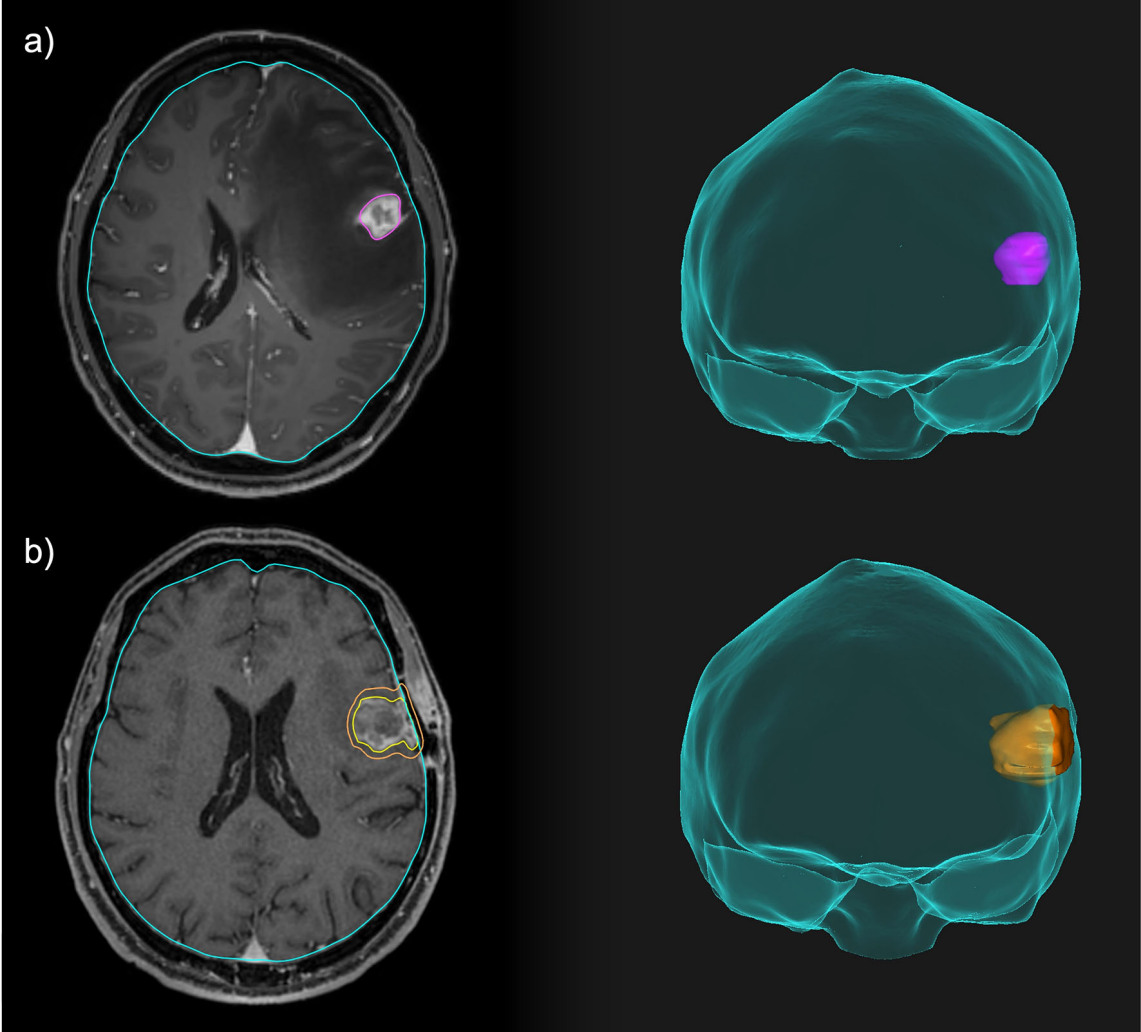
Comparison between neoadjuvant and adjuvant focal RT for a resectable brain lesion. **(A)** Pre-operative treatment volume including only the macroscopic disease. **(B)** Post-operative treatment volume including the resection cavity, the surgical tract and a 3-mm expansion margin.

**Figure 3:**
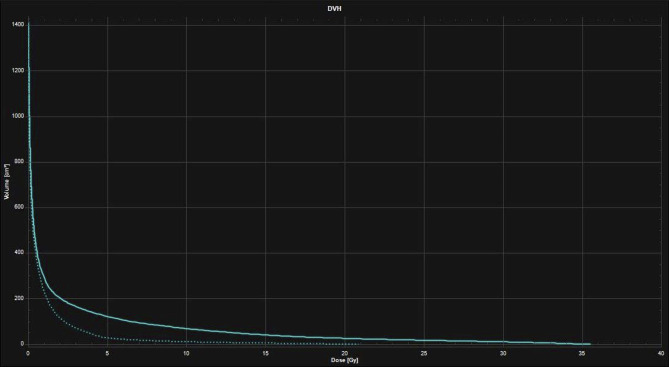
Dose-Volume Histograms (DVH) showing the lower dose received by the healthy brain with the 16 Gy neoadjuvant SRS treatment plan (dotted line) compared to the 27 Gy in 3 fractions adjuvant SRT treatment plan (continuous line).

Neoadjuvant SRS allows a better definition of the target, with a potentially reduced risk of LMD (sterilizing microscopic disease) and RN compared to the adjuvant setting. **Figures 2**, **3** illustrate volume and dose-volume histogram comparison between neoadjuvant and adjuvant focal RT.

Several single arm studies have collected retrospective and prospective data on neoadjuvant SRS, showing interesting efficacy and toxicity profile (including low rates of RN and LMD), but the small sample sizes are still inadequate to provide a robust evidence (51–56).

The recent PROPS-BM, a multicenter cohort study from 5 American institutions, represents the largest series of patients treated with neoadjuvant SRS to date. The outcomes of 242 patients (43.4% diagnosed with NSCLC) with 253 lesions (62.4% had a single BM) were analyzed. With a median time between SRS and surgery of 1 day (range 1-3 days), the median prescribed dose at the 80% isodose line was 15 Gy to a median GTV of 9.9 cm^3^. The treatment was effective (1-year LC: 85%; median survival time 16.9 months; LMD rate: 7.9%) and safe (postoperative complications: 7%; RN rate: 7.1%), with subtotal resection as strong independent predictor for local recurrence (57).

Ongoing studies, particularly the phase III trial from MD Anderson Cancer Centre (MDACC) (NCT03741673), Mayo Clinic (NCT03750227) and Alberta (NCT04474925), will provide further information on this promising approach **(****Table 1****)**.

**Conclusions:** For resectable BMs neoadjuvant SRS is a promising approach, with potential advantages if compared to adjuvant RT. Further evidence is needed for a greater implementation of this treatment into clinical practice.

## Oligometastases (2-4 LESIONS)

The oligometastatic brain disease represents a stage with limited number (usually up to 3-4) of BMs, expression of an intermediate status between the absence of brain lesions and a disseminated disease.

The distinction between oligo and polymetastic disease arises from the need for a simple system to categorize patients with BMs based on the number of lesions, in order to determine the appropriate therapeutic strategy. It is based on an empirical cut-off, extrapolated from the inclusion criteria of the main RCTs.

In this setting focal therapies determine excellent LC rates, without the same survival rates observed for the single lesion (9–11). Focal SRS represents the best solution to treat all the metastatic spots and to obtain the best oncological and clinical outcomes. Surgery plays a secondary role, and is usually limited to the resection of large and symptomatic lesions.

Historical data from the randomized RTOG 9508 trial showed a significant improvement in LC at 1 year (81% vs 71%, p=0.01) with the addition of SRS to WBRT in patients with 2-3 brain lesions, but OS did not change among the two groups. As an additional finding, patients with good prognosis (RPA I) showed a survival benefit with the combination of WBRT and SRS regardless of the number of lesions (11).

### SRS Alone With WBRT Omission

The demonstrated local efficacy of focal SRS has raised the question on the feasibility of omitting WBRT after SRS not only in patients with a single lesion but also in those with a limited number (2–4) of BMs, in order to keep WBRT as a salvage option and to delay or possibly avoid neurocognitive dysfunctions.

Four historical randomized trials and an individual patient data meta-analysis investigated this scenario with different endpoints (16–19, 22).

Overall, WBRT demonstrated an increase in local and distant intracranial control (by approximately 15-30% and 50% respectively), but without any survival benefit and at the price of a worsening in neurocognitive functions and QoL. These results generated a new trend to omit WBRT also in patients with few BMs, despite some controversial aspects.

In fact, it is known that intracranial relapse could be the primary cause of death in these patients, and WBRT has proved to be an effective strategy to achieve both local and distant intracranial control. At the same time, the progression of metastatic brain disease involving critical areas can cause rapid neurocognitive deterioration. The correct patient stratification, according to modern prognostic scores, could help to identify those patients that could benefit from the combination of WBRT and SRS in this peculiar setting.

With this purpose, 3 secondary analyses of the JROSG (58), EORTC (59) and Alliance (60) trials were conducted, post-stratifying the NSCLC populations according to the Disease Specific-GPA (DS-GPA). Among the 88 NSCLC patients of the Japanese study, better OS was observed with SRS + WBRT in the 47 with DS-GPA 2.5-4.0 (WBRT+SRS: 16.7 months vs SRS: 10.6 months, p = 0.04). No difference was observed in the unfavorable DS-GPA group (0.5-2.0), probably due to a less significant impact of intracranial control on patient’s prognosis (HR: 3.57 p =0.04 vs HR: 8.31 P < 0.001) (58). By contrast, the other two exploratory analyses of the EORTC and Alliance trials, did not demonstrate a survival benefit in favor of the addition of WBRT in any risk category according to DS-GPA (59, 60). **Table 2** resumes these findings.

**Table 2:** Secondary analyses of RCT post-stratifing NSCLC patients with oligometastases to the brain (1-3 or 4 lesions) according to DS-GPA.

Secondary analysis	Original trial	NSCLC analysed patients (Total number)	Main findings	Conclusions
Sperduto 2014	RTOG WBRT vs WBRT + SRS	211 (331)	GPA 3.5-4	WBRT + SRS in NSCLC oligometastases with favourable prognosis deserves further evaluation
			MST: WBRT + SRS 21.1 mo vs WBRT 10.3 mo (p=0.05)	
Aoyama 2015	JROSOG	88 (133)	DS-GPA 2.5-4	Consider WBRT in NSCLC oligometastases with favourable prognosis
	SRS + WBRT vs SRS		MST: SRS+WBRT 16.7 mo vs SRS 10.6 mo (HR=1.92, p=0.04)	
Churrilla 2017	EORTC	175 (329)	DS-GPA ≥ 2.5	Omit WBRT in NSCLC oligometastases undergoing SRS and close surveillance
	Focal thx + WBRT vs focal thx		OS: HR 1.11, p=0.641	
			DS-GPA< 2	
			OS: HR 1.10, p=0.690	
Churrilla 2017	Alliance	106 (213)	DS-GPA ≥ 2	Omit WBRT in NSCLC oligometastases undergoing SRS and close surveillance
	SRS + WBRT vs SRS		MST: SRS + WBRT 11.3 mo vs SRS 17.9 mo (p=0.63)	
			DS-GPA< 2	
			MST: SRS + WBRT 3.7 mo vs SRS 6.6 mo (p=0.85)	

GPA, Graded Prognostic Assessment; MST, Median Survival Time; WBRT, Whole Brain Radiation Therapy; SRS, Radiosurgery; DS-GPA, Disease Specific Graded Prognostic Assessment; OS, Overall Survival.

**Conclusions:** SRS alone could be safely administered to patients with 2-4 BMs. The omission of WBRT preserves the neurocognitive functions without compromising survival in the overall NSCLC population. Some secondary analyses suggest a potential survival advantage combining WBRT and SRS in NSCLC patients with 2-4 brain lesions and a favorable GPA score.

### Systemic Therapy in Oligometastatic Disease

Historically, systemic therapy played a secondary role in the management of BMs, considering the CNS as a “sanctuary site” for traditional chemotherapy (ChT) due to the blood brain barrier.

In NSCLC, the development of immune checkpoint inhibitors (ICIs) and targeted agents [particularly Epidermal Growth Factor Receptor (EGFR) and Anaplastic Lymphoma Kinase (ALK) Tyrosine Kinase Inhibitors (TKIs)], redesigned this scenario, for the ability of these novel drugs to permeate in the CNS. The new generation of targeted agents, such as osimertinib for EGFR-mutated (61, 62) and alectinib or lorlatinib for ALK-rearranged (63, 64) NSCLC, significantly improved intracranial response rate compared to traditional ChT or first-generation TKIs, with responses durable over time.

Today, these drugs may be integrated with focal therapies in a multimodal approach. The best sequence of treatment is not clear, and the choice must be individualized according to different clinical aspects, such as the time of BMs onset (synchronous vs metachronous), the time to previous treatments for oligoprogressive or oligorecurrent disease and the presence of neurologic symptoms. In patients with NSCLC and synchronous brain oligometastases, particularly with asymptomatic presentations, upfront systemic therapy is one of the most adopted strategy, deferring focal RT upon evaluation of clinical and radiological response.

In 2015 a Korean phase III trial evaluated the role of upfront ChT in de-novo oligometastatic disease from NSCLC. This study randomized 105 patients with 1-4 asymptomatic BMs to receive SRS + ChT vs ChT alone. Upfront ChT alone resulted in good response rates (37%) with no difference in intracranial control (median time: SRS 9.4 months vs ChT 6.6 months, p=0.248) and OS (SRS 14.6 months vs ChT 15.3 months, p=0.418) between the two arms (65). Similar studies with the use of new generation TKIs are expected to even improve these outcomes.

Another solution is the combined administration of SRS and targeted treatments. Data from retrospective series in oncogene-addicted NSCLC, preliminarily showed promising results in favor of the synergistic effect of this regimen in improving the intracranial disease control (66, 67) and this approach is under investigation in ongoing RCTs.

In patients with oligorecurrent or oligoprogressive NSCLC presenting metachronous BMs, the focal approach with SRS is even helpful to delay the start of a systemic treatment or the switch to a new regimen (66).

**Conclusions:** Upfront systemic therapy with the delay of RT administration is a viable option for asymptomatic synchronous brain oligometastases, particularly interesting in oncogene-addicted NSCLC. Conversely, focal RT can be the way to delay systemic treatment in case of metachronous oligorecurrence or oligoprogression to the brain.

## Polymetastatic Disease

The term polymetastatic refers to a clinical condition characterized by a significant number of BMs originating from a primary tumor mainly *via* the hematogenous route, which exposes the whole brain to the risk of micrometastatic disease. Nearly all published studies on polymetastatic disease have inclusion criteria which allow the enrolment of patients with >5 metastases. In this setting besides the number of BMs, other factors must be taken into account: age and performance status of the patient, volume of intracranial disease, histology, molecular biology, rate of progression of extracranial tumor burden (68). Current guidelines still maintain WBRT as the gold standard for the treatment of multiple symptomatic BMs; it is also strongly recommended in all situations in which the main objective is to prevent the onset of new metastases (69). WBRT was shown to improve neurological symptoms and function with minimal morbidity in this setting (70). It palliates symptoms, significantly improves intracranial control, and reduces the risk of neurological death (58, 71). In historical studies, WBRT increased median OS up to 3 to 6 months if compared to simple observation (70, 72) with an Overall Response Rate (ORR) ranging from 64% to 85% (70, 73).

In NSCLC patients with less than 3 months life expectancy and/or poor performance status, optimal supportive care (OSC) with corticosteroids is a reasonable alternative compared to WBRT as revealed by the phase III non-inferiority Medical Research Council trial (QUARTZ). This study compared OSC alone and OSC plus WBRT in NSCLC patients with BMs not amenable to surgery or SRS, reporting similar QoL at 4-, 8- and 12-weeks and OS (HR 1.06, p=0.8084) in the two arms. The median survival was 9.2 weeks for patients who received OSC plus WBRT and 8.5 weeks for patients who received OSC alone. Anyway, after stratification for prognostic factors, patients aged younger than 60 years were shown to have a better OS with WBRT (p=0.0062). A similar trend was observed for patients with ≥ 5 metastases, good GPA class and KPS ≥ 70 (74).

WBRT is currently the most widely used option in clinical practice worldwide for multiple BMs (69, 75). From a technical point of view WBRT is a relatively simple RT technique (opposed laterals fields) which involves the irradiation of the whole brain and of the meninges. Moderately hypofractionated schedules are employed in order to reduce overall treatment time and to improve patients’ compliance. The WBRT prescription of 30 Gy in 10 fractions is universally accepted; other schedules include 20 Gy/5 fx, 37.5 Gy/15 fx and 40 Gy/20 fx.

Cognitive deterioration is a major complication of WBRT, with severe dementia that can appear several months to years following cranial irradiation. However, according to recent clinical evidence neurocognitive impairment may arise early on, with a component of short-term neurocognitive decline that may occurs within the first 1-4 months (17, 76, 77).

The following section will provide an outline of currently available therapeutic options and future perspectives aiming to reduce or prevent these complications and to simultaneously increase local tumor control.

## How to Limit Neurocognitive Impairment

### Neuroprotectors

The irradiation of the brain is associated with a dose-dependent radiation-induced leukoencephalopathy as a result of demyelination, vascular compromise and direct damage to neurons (78, 79). Patients affected by leukoencephalopathy may develop some degree of cognitive dysfunction which can compromise the QoL and affect memory, executive function, attention and concentration, and could lead to learning disorders and dementia (80). This entity is associated with diffuse supra-tentorial white matter abnormalities and cerebral atrophy. Apart from leukoencephalopathy, cranial irradiation can damage the hippocampus which has a fundamental role in the memory function.

The use of a tumor-selective agents that enhances the effects of radiation in tumors but spares normal brain tissue might extend the therapeutic ratio of WBRT, improving LC without increasing radiation toxicity.Certain agents target tumors selectively, generating reactive oxygen species intracellularly and lowering the apoptotic threshold to radiation and chemotherapy (81).

One of the most accredited theories suggests vascular damage and mineralizing microangiopathy, with subsequent small vessel insufficiency and infarction as it is seen in vascular dementia, as the leading cause of RT-related neurotoxicity (82, 83). Therefore, neurotransmitter regulators commonly used to treat vascular dementia such as memantine have also been taken into consideration. In the RTOG 0614, a placebo double-blind RCT, the use of daily memantine for 24 weeks during and after WBRT resulted in better cognitive function over time, delayed time to cognitive and memory decline, executive function, and processing speed compared to placebo (84).

A recent drug, donepezil (an acetylcholinesterase inhibitor), is being tested in a phase 3 study among long-term adult brain tumor survivors after a course of fractionated WBRT or PCI, with the aim of improving cognitive impairment associated with brain cancer and its treatments. Despite modest improvements in several key cognitive functions, especially among patients with greater pre-treatment cognitive impairment, treatments with donepezil did not significantly improve the overall composite score (85).

The addition of Motexafin Gadolinium (MGd) to WBRT did not produce a significant overall improvement compared to WBRT alone (86). Anyway, in the intent-to-treat analysis, MGd exhibited a favourable trend in neurologic outcomes, significantly prolonging the interval to neurologic progression in NSCLC patients with BMs receiving prompt WBRT (87). Other molecules have been tested but promising results have been obtained for other histologies (88) and not for NSCLC.

Preliminary studies have demonstrated that BMX-001 provides protection of normal tissues from radiation-induced injury. Ongoing clinical trial (NCT03608020) will provide information on safety, tolerability and neurocognitive preservation of this drug.

**Conclusions:** Of the several molecules available to limit neurocognitive toxicity, only memantine prolongs time to neurocognitive and neurologic progression in patients with BMs from NSCLC.

### Hippocampal Avoiding-WBRT (HA-WBRT)

As anticipated, the hippocampus has an important role in learning, memory and mood regulation (89). Preclinical studies have demonstrated that relatively modest doses of radiation cause an early and significant decline in neurogenesis in the subgranular zone of hippocampi associated with suppression of new memory formation and impaired recall (90). In addition, recent clinical studies have observed a dose-response related risk of postradiotherapy decline in learning delayed recall caused by the dose of radiation absorbed by the hippocampus (91, 92). More in general, cranial irradiation plays a crucial role for memory decline. Memory function, specifically recall and delayed recall, as assessed with the Hopkins Verbal Learning Test Revised (HVLT- R), have a statistically significant neurocognitive decline at 4 and 6 months from WBRT (17), with a simultaneous impairment of patient-reported QoL (93).

**Figure 4:**
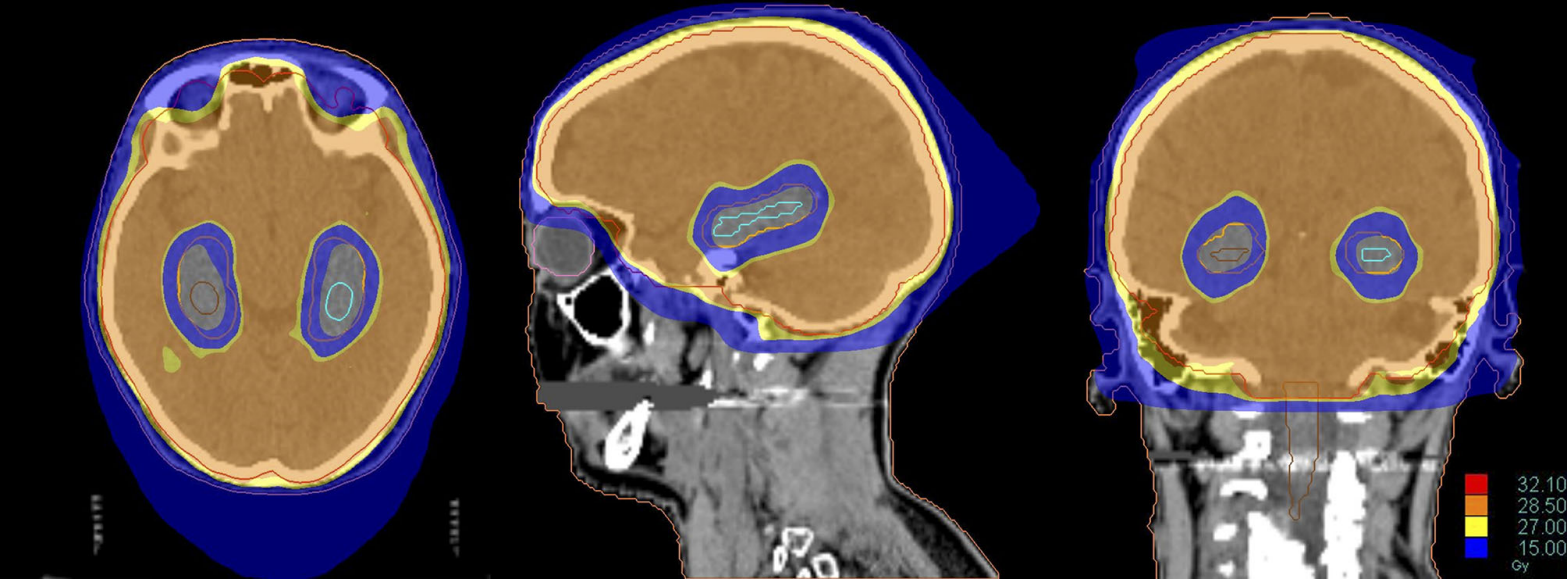
Dose distribution of a Hippocampal Avoidance WBRT (HA-WBRT) in Helical Tomotherapy. The prescription dose was 30 Gy/10 fx with hippocampal sparing (outlined on the right in brown and on the left in light blue, with an expansion margin of 5 mm corresponding to the PRV).

Recent technological improvements in radiotherapy, including helical tomotherapy, LINAC-based IMRT or volumetric-modulated arc therapy (VMAT), may be adopted to selectively spare anatomical structures involved in memory and learning during cranial irradiation (**Figure 4**) (92, 94).

**Figure 5:**
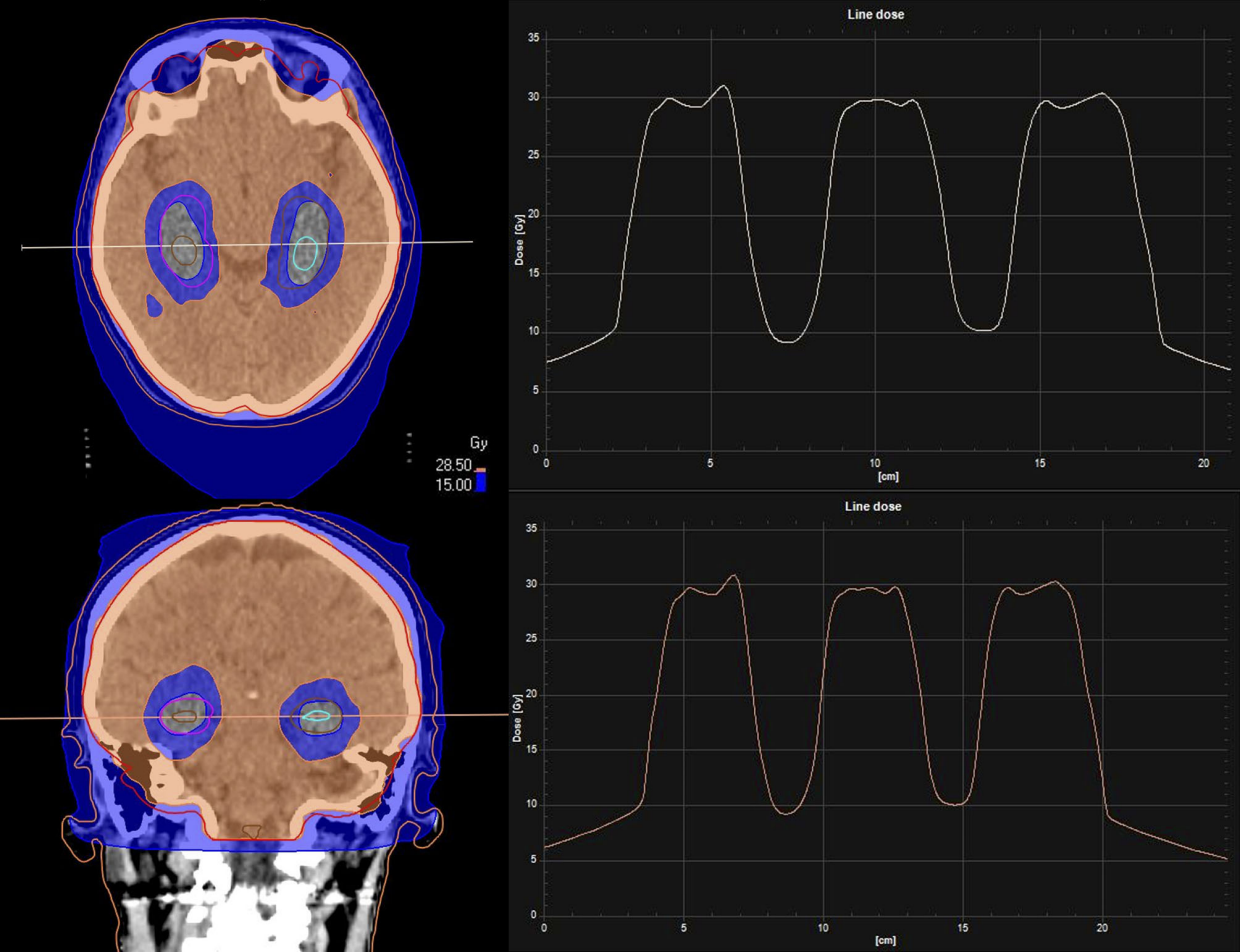
Dose line of HA-WBRT: the dose line shows the drop in dose at the level of the hippocampi, and the rapid rise to the prescription dose of 30 Gy/10 fr in the surrounding areas included in the CTV of the WBRT.

Gondi et al. first proved that HA-WBRT is able to reduce both maximum and mean dose per fraction delivered to the hippocampus (94). For a prescription dose of 30 Gy in 10 fractions to the whole brain, HA-WBRT is able to reduce the mean dose per fraction to the hippocampus (normalized to 2-Gy fractions) by 87% using helical tomotherapy and by 81% using LINAC-based IMRT. The maximum dose received by the hippocampus is 12.8 Gy (Dmean 5.5 Gy) using helical tomotherapy, and 15.3 Gy (Dmean 7.8 Gy) using LINAC based IMRT, with acceptable target coverage and homogeneity (94) (**Figure 5**).

On the basis of these feasibility analyses, randomized prospective trials were designed to prove the efficacy of HA-WBRT as a viable option for polymetastatic disease. The single-arm phase II RTOG 0933 trial on HA-WBRT for BMs reported significant memory preservation (assessed through reduction of HVLT-R Delayed Recall decline), compared with historical data of patients treated with standard WBRT (92). Further confirmation of the effectiveness of HA-WBRT came from a prospective randomized phase III trial (NRG-CC001) which evaluated the potential combined neuroprotective effects of hippocampal avoidance in addition to prophylactic memantine during WBRT. HA-WBRT + memantine prolonged time to neurocognitive failure; decrease in neurocognitive function at 6 months was 59.5% vs. 68.2% (HR: 0.76, p = 0.03) favouring the combination of HA-WBRT + memantine, without any difference in intracranial recurrences (p = 0.208) or OS (p = 0.307) (95).

Ongoing research on the pathophysiology of brain irradiation damage may reveal other possible important brain structures or hippocampal subregions (e.g. cornu ammonis) whose sparing could also contribute to better preservation of neurocognitive function (NCT04801342, NCT03223922).

It should be noted that sparing the hippocampus and the peri-hippocampal region determines the theoretical risk of intra-cranial disease progression/relapse in these anatomical regions. However, it is estimated that this volume accounts for approximately 2% of the whole brain and the incidence of the development of metastases in this area is an uncommon event with a detection rate of less than 10% in previous reports (94).

**Conclusions:** HA-WBRT is an alternative solution and should be considered in selected patients with good KPS to better preserve cognitive function and patient-reported symptoms. The combination with memantine proved to improve the neurocognitive endpoints in a RTOG trial and is now approved in the United States in combination with HA-WBRT.

### Focal Treatments as an Alternative to WBRT

Although current evidence confirms WBRT as the standard treatment for patients with multiple BMs (75), focal irradiation is increasingly used in daily clinical practice and many institutions extend its utilization beyond the oligometastatic setting, mostly in fear of the neurocognitive side effects of WBRT. It remains controversial which subpopulation of multiple BM patients may obtain the greater benefit from local treatments like SRS. In published series, predictive factors for LC comprise delivered dose, total volume of treated metastasis, and histology of the primary tumor (96–100) without a clear correlation with the total number of BMs. To date, only two prospective studies have been published. Thus, clinical data is currently quite limited. The multicenter phase 2 study by the Japanese Leksell Gamma Knife Society (JLGK0901) reported the outcome of patients treated with SRS alone upfront, regardless of the number of BMs, and reported the significant inferiority in OS for patients with 5-10 BMs (largest tumor <10 mL in volume and <3 cm in longest diameter; total cumulative volume ≤15 mL), compared with patients with only 2-4 BMs, although the difference in median survival time among the two groups was not clinically meningful (7.0 vs 7.9 respectively). On the other hand, cumulative incidences of neurological death at 6, 12, and 24 months after SRS did not differ significantly between patients with 2-4 BMs, and those with 5-10 BMs, nor did the cumulative incidence of neurological deterioration after SRS (11.6% vs 13%, p=0.54). At 12 months after SRS, neurocognitive function was maintained in 91% of patients with 2-4 BMs and 88% of patients with 5-10 BMs (p=0.60) (101).

Subsequently, Yamamoto et al. conducted a dedicated case-matched study comparing SRS in patients with 2–9 BMs and in those with 10 or more BMs. In this study, median survival time for the two groups was not significantly different, likewise neurological death-free survival, cumulative incidence of local recurrence, use of salvage SRS for new lesions, neurological deterioration and SRS-related complications. They concluded that even patients with 10 or more BMs may be suitable candidates for SRS after careful selection (e.g., low intracranial tumor burden) (100). A multicentre, single-arm, phase 2 study by Nichol et al. also reported the effectiveness and tolerability of SRS for patients with 1 to 10 BMs (102).

Other retrospective studies have confirmed SRS as appropriate in patients with polymetastatic disease with LC rate and toxicity comparable to those observed in patients with a limited number of BMs (103–105).

Recently, the first phase III randomized controlled trial (Dutch Trial) investigating WBRT versus SRS for patients with 4–10 BM suggested that SRS is a valid palliative treatment option for patients with polymetastatic disease for the reduced incidence of toxicity and for the preservation of QoL. Anyway, the trial was closed prematurely, mainly as a result of patients’ and doctors’ preference for SRS, and no definitive results were produced on the non-inferiority of SRS in term of OS and brain failure-free survival (106).

Ongoing phase III trials directly comparing WBRT to SRS in patients with multiple BMs will hopefully bring more relevant data and evidence to guide the treatment selection.

A randomized phase III trial at the MDACC has randomized 72 patients with 4–15 BMs from a non-melanoma primary tumor to SRS (15-24 Gy) or WBRT (30 Gy) (107). The results presented at the annual ASTRO meeting in 2020 showed similar LC, new onset of BMs and OS (approximately 8 months) among the two cohorts, while patients receiving SRS had a shorter time to systemic therapies (2 weeks vs 4) and a better preservation of neurocognitive function. A clinical trial led by the National Cancer Information Center, is randomizing patients with 5-15 BMs to SRS or WBRT in combination with memantine. OS and neurocognitive deterioration free survival are the primary endpoints (NCT03550391). Another trial from Dana-Farber Cancer Institute (NCT03075072) is randomizing patients with 5-20 BMs to SRS or WBRT plus hippocampal sparing and QoL is the primary endpoint (108). **Table 3** resumes the ongoing studies investigating this innovative setting.

**Table 3:** Currently ongoing clinical trials investigating SRS/SRT for multiple BMs.

NCT identifier	Study phase	Number of patients estimated	Study population	Standard arm	Experimental arm	Primary endpoint
***Focal treatment as an alternative to WBRT***						
NCT02953717 (Elisabeth-TweeSteden Hospital Tilburg)	NA	80	BMs 11-20	WBRT	Multiple SRS	Cognitive decline at 3 months
NCT04891471 (Mediterranean Institute of Oncology)	NA	100	BMs > 5	WBRT	SRS/SRT	NCF changes, changes of autonomy in daily activities, change in QoL
NCT03775330 (Sunnybrook Odette Cancer Centre Toronto)	NA	125	BMS: 5-20	–	A. : SRSB. : SRS + WBRT	NCF
NCT03075072 (Dana-Farber Cancer Institute)	III	196	BMs 5-20	WBRT	Multiple SRS	QoL
***Focal treatment as an alternative to HA-WBRT***						
NCT03550391 (Canadian Cancer Trials Group)	III	206	BMs: 5-15	–	A. : HA-WBRT + MemantineB. : SRS	OS, Neurocognitive PFS
NCT04277403 (Medical University of Innsbruck)	III	150	BMs:4-15	–	A. : HA-WBRT+SIBB. : SRS/SRT	iPFS

NA, not applicable; WBRT, Whole Brain Radiation Therapy; SRS, Radiosurgery; SRT, fractionated stereotactic radiotherapy; QoL,Quality of Life; NCF,NeuroCognitive Function; HA-WBRT, Hippocampal Avoidance-WBRT; PFS, Progression Free Survival, iPFS, Progression Free Survival.

**Conclusions:** WBRT remains the standard treatment for patients with >4 metastases. However, there is growing evidence to support the role of SRS for patients with 4-15 metastases to improve the risk-benefit ratio of these patients, reserving WBRT as salvage treatment in case of rapid and progressive intracranial disease. It remains controversial which subpopulation of multiple BM patients benefits most from local treatments, including SRS.

## How to Improve Local Control

### WBRT+Simultaneous Integrated Boost (SIB)

In the past, dose escalation with WBRT plus sequential boost was employed to improve LC within the context of oligometastatic disease (11, 16).

The new technical frontier is to combine WBRT with a simultaneous integrated boost (SIB) in patients with multiple BMs. Modern RT techniques, such as dynamic IMRT and VMAT, are able to generate steep dose gradients between close volumes and to simultaneously delivery different doses to the whole brain and to visible BMs. SIB is then the technological evolution of the sequential boost, with several technical and logistical advantages. SIB deliveries/includes WBRT and a boost on visible BMs in the same session, with an optimized dose distribution, a single simulation protocol, an improvement in patients’ compliance and a reduction in overall treatment time and costs (109). The first feasibility studies involved patients with a limited number or volume of BMs (110, 111). The same results emerged even in patients with a larger number and volume of BMs (109, 112). WBRT+SIB on large lesions appears to be safe and effective even for patients with 4-10 BMs, without significant cognitive decline (113); therefore, SIB is frequently employed for the treatment of poly-metastatic disease. Recently it has also been shown to provide a significantly longer median intracranial PFS (9.1 vs 5.9 months, p=0.001) and median OS (14 vs 11 months, p=0.037) compared to WBRT + sequential boost (114, 115). There is no consensus on the most appropriate hypofractionation schedule and each institute bases the choice on clinical judgment and experience. The most frequently used schedule is in 5 daily fractions with a dose of 20 Gy (4 Gy per day) to the whole brain and a SIB of 40 Gy (8 Gy per day) to the visible BMs (110).

**Figure 6:**
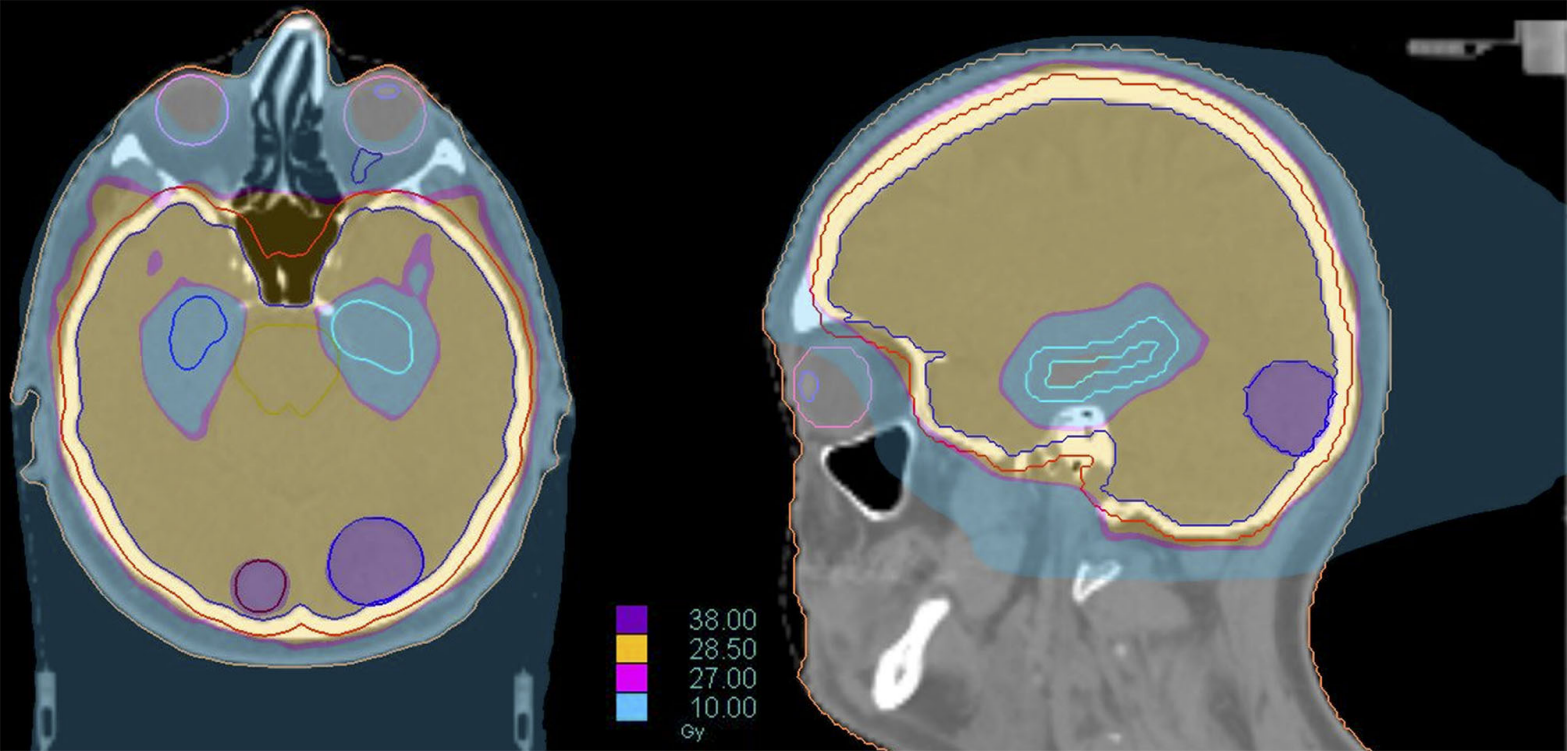
Dose distribution of a treatment of HA-WBRT plus SIB in patient with bilateral occipital BMs (left 2 cm; right 7 mm). The treatment was delivered with Helical tomotherapy and a daily control of the patient’s positioning with MV-CT. The dose delivered to the whole brain was 30 Gy in 10 fx with a concomitant boost of 40 Gy in 10 fx to the 2 lesions.

Modern technologies allow for a further innovative step, represented by the addition of hippocampal avoidance in this integrated approach (HA-WBRT+SIB). Through a VMAT or, even better, helical tomotherapy planning, it is possible to simultaneously spare the hippocampi, deliver high doses of radiation to multiple metastases and treat the remaining brain volume with a homogeneous dose distribution, all in a single treatment plan/session (116, 117) (**Figure 6**). HA-WBRT+SIB can be an effective therapeutic option for patients with multiple BMs: it shows improved LC of treated metastases (98% vs 82% at 1 year; P = 0.007) and improves overall intracranial PFS in comparison with WBRT alone (13.5 vs 6.4 months; P = 0.03) (118).

The potential of HA-WBRT+SIB to prevent neurocognitive effects and to concomitantly improve LC is currently under investigation in randomized, multicenter trials (119, 120).

**Conclusions:** WBRT+SIB improves LC for the treatment of multiple BMs. The combination of HA-WBRT with SIB could reduce deterioration in neurocognitive function and further improve the therapeutic index for these patients.

## Leptomeningeal Dissemination

Leptomeningeal dissemination (LMD), also known as leptomeningeal carcinomatosis, is a rare complication occurring in ∼10% of patients with metastatic cancer, but is particularly frequent in patients with BMs originating from NSCLC (56-82% of cases), especially for adenocarcinomas (84-96%) (121). Neurosurgery may favor tumor cell spreading and therefore the incidence of LMD is higher in patients treated with surgery than in patients treated with upfront SRS (122). Without treatment, the median survival is dismal (6–8 weeks) in NSCLC patients, whereas survival may be prolonged beyond 6 months with treatment, including targeted therapy (123–125) and immunotherapy (126), with a 1-year OS rate of 19% (127, 128). LMD is frequently associated with moderate to severe neurological symptoms and the aim of treatment is to prolong survival while maintaining an acceptable QoL and delaying neurological deterioration.

There is no consensus regarding the optimal treatment for patients with LMD and recommendations for the treatment modalities are supported by a low level of evidence (129), as no randomized trial is currently available.

Diagnosis and management of patients with LMD should follow a multidisciplinary discussion.

**Figure 7:**
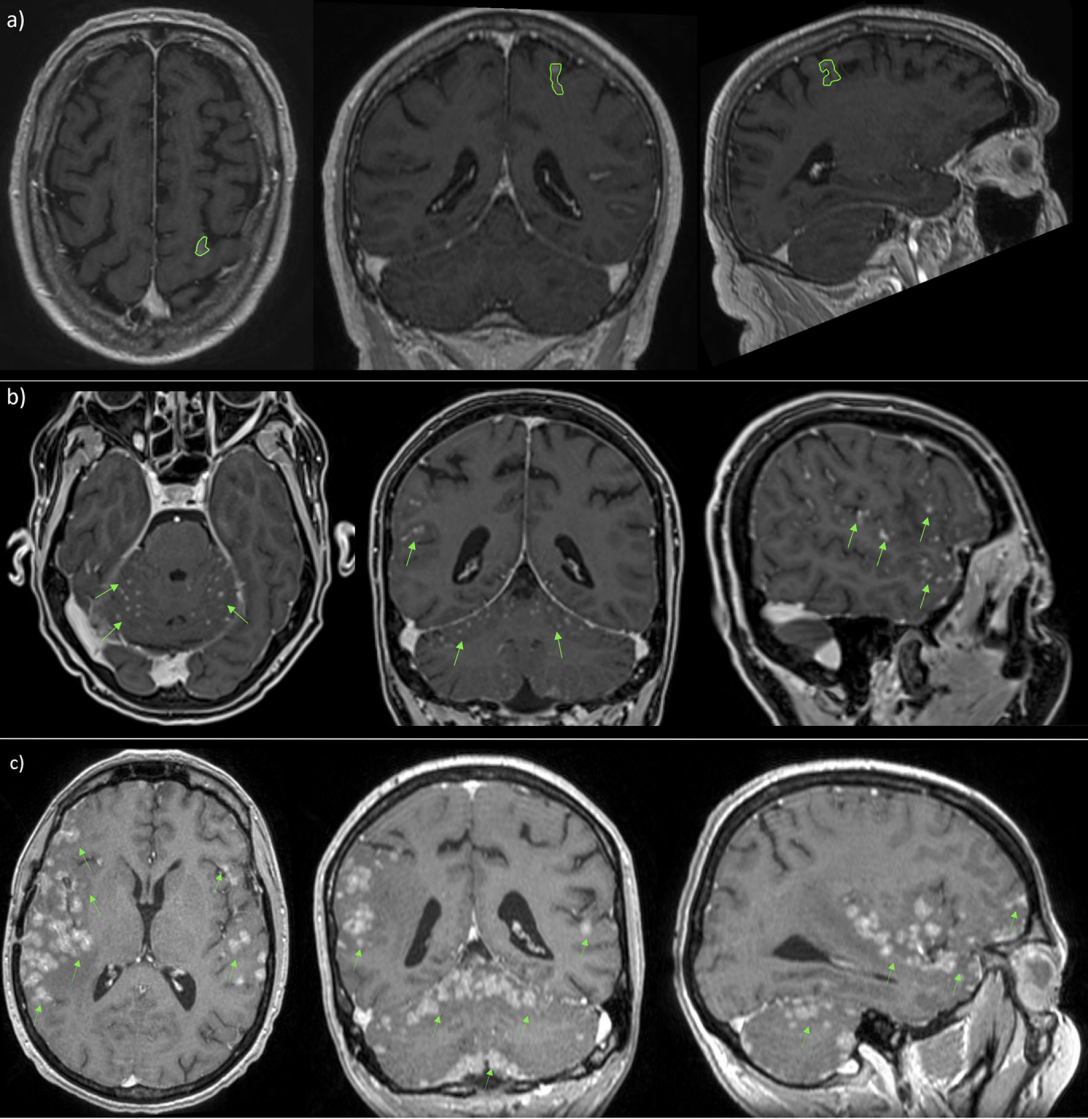
MRI scans in patient with nodular **(A)**, linear **(B)** and both types **(C)** of leptomeningeal metastasis.

Recently, a new radiological classification of LMD, based on MRI findings, is being developed in order to guide the selection of the optimal therapeutic strategy. It identifies linear LMD (type A), nodular leptomeningeal disease (type B), both (type C), no neuroimaging evidence of LMD or, at least, hydrocephalus (type D) (121, 130) (**Figure 7**).

Current management approaches include a range of treatments such as systemic ChT for the primary tumor and extra-CNS disease (preferred choice for type B/C LMD), intrathecal ChT targeted therapy, RT, and supportive care (131).

In particular, the utility of WBRT for LMD has been evaluated retrospectively by Hirano and colleagues. The authors demonstrated that WBRT (30 Gy/10 fx) was effective for the relief of symptoms but provided only little benefit in these patients expected to have poor survival (132).

The NCCN has recently incorporated risk stratification and guidelines for the treatment of LMD. RT is typically included in the multimodal treatment of patients with Good Risk LMD, namely those with a KPS ≥60, the absence of major neurologic deficits, the presence of minimal systemic disease or the opportunity to start/prosecute a systemic treatment (12).

In current clinical practice, focal RT administered in fractionated regimens such as involved-field RT or SRT or SRS, can be used to treat nodular disease and symptomatic cerebral or spinal lesions. In rare cases, focal RT can be employed for cauda equina syndrome or cranial nerve palsy after exclusion of other causes, even in the absence of corresponding MRI findings.

Since the presence of cerebro-splinal fluid (CSF) flow interruptions is associated with decreased survival, normal CSF flow can be restored with focal RT in 30% of patients with spinal blocks and in 50% of patients with intracranial blocks, and has been proposed to reduce the toxicity, and enhance the efficacy of intra-CSF therapy (121). Typical target volumes for RT in the presence of cranial neuropathies include the base of cranial floor, the interpeduncular cistern and the first two cervical vertebrae, while in the presence of a cauda equina syndrome the lumbosacral vertebrae are also included.

WBRT may be a valid option in extensive nodular LMD, symptomatic linear LMD or coexisting BM.

Given the high rate of toxicity (bone marrow toxicity, enteritis and mucositis) and the usual co-existence of systemic disease, craniospinal RT, especially in combination with systemic or intra-CSF treatment, is rarely employed in reason of its severe toxicity.

A recent report by Prabhu et al. found that nodular LMD was a distinct pattern of LMD, associated with surgery and postoperative SRS, that was less likely to be symptomatic and had better OS rates than classical linear (“sugarcoating”) LMD (130).

Furthermore, when patients with LMD were treated with focal RT (SRS or partial brain), they had a higher risk of LMD recurrence, but no detrimental effect on OS compared with WBRT-containing regimens, which means focal RT provides a reasonable, less toxic, treatment strategy for these patients (131).

In conclusion, there is limited high-quality evidence to guide the optimal use of RT for the treatment of LMD, and there is a great need for prospective trials. Furthermore, in this changing environment with constant advancements in diagnostic procedures and in systemic therapy (targeted therapy and immunotherapy), the role of RT will likely continue to evolve and advance.

**Conclusions:** WBRT remains the standard treatment in case of LMD. Despite limited high-quality evidence, focal RT could be considered for localized lesions, especially if symptomatic.

## Combined Modality Treatment: RT and Systemic Therapy

The increasing use of focal brain radiotherapy (SRS/SRT), even when dealing with multiple BMs, has led to consider the possibility of combining these techniques with systemic treatments, in order to exploit any potential synergic effect of “chemo-immuno-radiation” (133). Despite the various studies aimed at examining this combination various issues remain uncertain, including the potential toxicities of these associations (134).

In particular, there is lack of consensus regarding the ideal timing for RT and the need for a washout when a patient is receiving a systemic treatment. A recent review included 6384 patients and analyzed the toxicity of concurrent SRS and ChT, immunotherapy (IT), and/or targeted therapies (TT). Despite the heterogeneity of the studies included in this evaluation, the authors concluded that SRS combined with systemic therapy appears to be safe, with no significant increase of side effects (major bleeding, RN, skin toxicity), allowing the maintenance of systemic agents during SRS (135).

Cho et al. presented similar results for patients receiving Gamma knife SRS concurrently with IT or TT (136). They observed no statistically significant differences in the occurrence of RN, or intralesional hemorrhage in association with IT or TT during or after SRS.

### Radiotherapy and Targeted Therapy

Early studies investigating the RT-TKI combination have compared patients treated with WBRT + erlotinib or WBRT alone, revealing a statistically significant increase in ORR, median PFS and median OS (137, 138). These results were not confirmed in a recent phase III trial, showing that concurrent erlotinib with WBRT does not improve intracranial PFS or OS for NSCLC patients with BM, but the combination therapy is well tolerated with no unexpected neurotoxicity (139). A retrospective study by Chen et al, comparing the combination of EGFR-TKI and RT (WBRT and SRS) and EGFR-TKI alone, revealed no statistically significant differences in PFS and OS but only in median time to intracranial progression (21.5 vs 15 mo, p=0.036) (140). In view of the inconclusive data published until now, ongoing prospective trials are examining the effects of the SRS/EGFR-TKI combination in patients with known EGFR-status (**Table 4**).

**Table 4:** Selected ongoing clinical trials investigating the combination of target therapies and RT in NSCLC patients with BMs.

NCT identifier	Study phase	Number of patients estimated	Study population	Standard arm	Experimental arm	Primary endpoint
NCT03535363 (Case Comprehensive Cancer Center)	I	6 (actual enrolment)	Stage IV EGFR Mutated with 1-10 BMs	–	Osimertinib before, concurrently and after SRS	MTD of Osimertinib with SRS
NCT04147728 (Peking University Third Hospital)	II	50	Limited BMs (1-5)	–	Anlotinib + SRS	Edema Index
NCT04905550 (Chongqing University Cancer Hospital Chongqing)	II	50	Stage IV EGFR Mutated with BMs	–	Almonertinib + SRT or SRS or WBRT	iPFS
NCT03769103 (British Columbia Cancer Agency)	II	76	Stage IV EGFR Mutated with BMs	SRS + Osimertinib	Osimertinib alone	iPFS
NCT03497767 (Trans Tasman Radiation Oncology Group)	II	80	EGFR-mutated NSCLC with BMs diagnosed *de novo* or developed while on first-line EGFR-TKI	–	A. : Osimertinib aloneB. : Upfront SRS followed by Osimertinib	1y iPFS
NCT04829019 (Department of Medical Oncology, Cancer Center of Sun Yat-Sen University Guangzhou)	II	88	EGFR-mutated NSCLC with BMs	Osimertinib + WBRT	Osimertinib	NCF
NCT02726568 (West China Hospital Chengdu)	II	30	EGFR-mutated NSCLC with BMs	–	Icotinib + SRS when intracranial progression	iPFS
NCT03754530	II	162	EGFR-mutated NSCLC with BMs	–	A. : IcotinibB. : Icotinib + RT (WBRT or SRS)	iPFS
NCT04193007 (The Second Affiliated Hospital of Nanchang University Nanchang)	II	100	Asymptomatic NSCLC BMs with Gene-Sensitive Mutation	Molecular targeted therapy (EGFR-TKI or the first generation of ALK inhibitors)	Brain Radiotherapy and molecular targeted therapy	iPFS, ORR
NCT04058704 (Zhejiang Cancer Hospital Hangzhou)	III	296	EGFR-mutated NSCLC with BMs	–	A. : Early intervention (Icotinib + RT)B. : Late intervention (Icotinib + RT	OS
NCT02714010 (Sun Yat-sen University of cancer center Recruiting Guangzhou)	III	601	Stage IV EGFR Mutated with BMs	EGFR-TKI + concurrent WBRT	EGFR-TKI alone till tumor progression	iPFS
NCT02882984 (Sichuan PPH, Cancer Center Recruiting Chengdu)	III	325	Stage IV EGFR Mutated with BMs	WBRT along with TKI	HFSRS with EGFR TKI	iPFS

NA, not applicable; WBRT, Whole Brain Radiation Therapy; SRS, Radiosurgery; SRT, fractionated stereotactic radiotherapy; EGFR, Epidermal Growth Factor Receptor; TKI, Tyrosin Kinase Inhibitor; iPFS, Progression Free Survival; MTD, Maximum Tolerated Dose; NCF,NeuroCognitive Function; OS,Overall Survival; ORR, Overall Response Rate.

Regarding the association of ALK inhibitors and RT, the combination of crizotinib and brain RT brought a statistically significant increase in ORR and median time to tumor progression (7 vs 13.2 months, respectively) (141). Another cohort of ALK-rearranged NSCLC patients treated with RT and TKIs showed favorable intracranial PFS and OS rates (142). However, the ASCEND-4 trial presented no statistically significant differences in terms of outcomes for the addition of RT to ceritinib (143). No relevant results and acute toxicity data are available to justify the implementation in clinical practice of the combination ALK-TKI- RT. To date most of the ongoing trials evaluate the combination EGFR-TKI- RT.

**Conclusions:** The combination of EGFR and ALK TKIs + RT is still under investigation and preliminary results suggest a possible benefit, particularly in terms of intracranial control, regardless of the radiotherapy technique (SRT or WBRT) and number of brain lesions. The ideal timing for the combination, the type of RT (focal/WBRT) and possible toxicities must be investigated.

### Radiotherapy and Immunotherapy

The discovery of a lymphatic drainage system in the brain and the ability of T-cells to cross the blood-brain barrier has led to believe that the combination of RT and IT may improve antigen presentation in T-cells (144).

Data regarding the IT-RT combination in driver negative NSCLC patients, mostly from retrospective studies, is contradictory.

Clinical data revealed an increase in OS for the combination of RT and ICIs, starting immune checkpoint inhibitors (ICIs) at least 30 days earlier and continued throughout the RT treatment (145).

The retrospective study by Ahmed et al. presented no additional toxicity for NSCLC patients with BMs who received SRS and anti-PD1/PDL1 therapy (146).

So, there is reassuring data regarding the safety profile and efficacy of the combination of anti-PD1/anti-PDL1 agents and various RT regimens (SRS, WBRT) (147), with only a potential warning on an increased risk of RN (M155). In general practice, at least 50% of physicians do not interrupt ICIs when patients require SRS or WBRT (148).

A randomized study on ICIs with or without SRS in patients with asymptomatic BMs is still lacking to date. The **Table 5** shows various ongoing trials examining the use of IT together with different brain radiation techniques.

**Table 5:** Selected ongoing clinical trials investigating the combination of Immunotherapy and RT.

NCT identifier	Study phase	Number of patients estimated	Study population	Standard arm	Experimental arm	Primary endpoint
NCT04047602 (Indiana University Health Hospital Recruiting Indianapolis)	NA	42	BMs (1-10) from NSCLC	–	Reduced Dose SRS based on the brain tumor size concurrently with standard of care IT	Symptomatic RN rate
NCT03458455 (Oslo University Hospital Oslo, Norway)	NA (Cohort, Prospective)	200	BMs from NSCLC, BMs from malignant melanoma	–	A. : BMs from NSCLC receiving SRS to selected lesionsB. : BMs from malignant melanoma receiving SRS to selected lesionsC. : BMs from NSCLC receiving SRS to selected lesions + nivolumab or pembrolizumabD. : BMs from malignant melanoma receiving SRS to selected lesions + ipilimumab, nivolumab or pembrolizumabE. : BMs from NSCLC receiving SRS to selected lesions + EGFR inhibitors	Treatment Response
NCT04787185	NA (Multicenter, Prospective Observational Study)	50	BMs from NSCLC	–	SRT + IT	Evaluation of toxicity
NCT02858869 (Emory University/Winship Cancer Institute Atlanta)	I	30	Stage IV NSCLC and melanoma	–	A. : Pembrolizumab, SRS 6 Gy x 5 fx, ClosedB. : Pembrolizumab, SRS 9 Gy x 3 fxC. : Pembrolizumab, SRS 18-21 Gy	Safety of 3 different SRS radiation arms in combination with pembrolizumab
NCT02696993 (M D Anderson Cancer Center Houston)	II	88	Stage IV NSCLC	–	A. : Nivolumab, SRSB. : Nivolumab, WBRTC. : Nivolumab, Ipilimumab, SRSD. : Nivolumab, Ipilimumab, WBRT	RP2D of Nivoluma, RP2D of Nivolumab + Ipilimumab, RP2D of Nivolumab + SRS/WBRT, RP2D of Nivolumab + Ipilimumab and SRS/WBRT
NCT02978404 (Centre Hospitalier de l’Université de Montréal (CHUM) Montreal)	II	60 (26 actual enrollment)	Stage IV NSCLC, SCLC, Melanoma OR ccRCC	–	SRS and Nivolumab	iPFS
NCT04427228 (University Of Chicago Chicago)	II	74	BMs from different histology in patients treated with immunotherapy (PD-1/PD-L1 and/or CTLA-4 inhibitor(s)) within the past 6 months or plan on receiving immunotherapy within the next 1 month.	SRS (20 or 18 Gy)	Radiosurgery Three Treatments (27 Gy/3fx)	Multi-Fraction SRS superiority compared to single fraction SRS
NCT04650490 (Duke Cancer Center Durham)	II	80	BMs (1-15) from NSCLC	–	A. : Immediate SRS followed by ITB. : Immediate IT followed by SRS	iPFS
NCT04889066 (University of Texas Southwestern Medical Center)	II	40	BMs from NSCLC	Durvalumab + standard SRT	Durvalumab + PULSAR (Personalised Ultra fractionated Stereotactic Adaptive Radiotherapy)	Intercranial clinical benefit
NCT04291092	II	20	BMs from NSCLC	–	SHR-1210 + WBRT/SRS	PFS, ORR
NCT04180501 (Union hospital Wuhan, Hubei, China)	II	25	BMs from advanced NSCLC	–	SRS sequential sindilimab	iPFS
NCT04768075 (Guangdong Association of Clinical Trials)	III	200	BMs (≥ 3) from driven gene-negative NSCLC	Placebo combined with chemotherapy (pemetrexed or paclitaxel or Nab-paclitaxel + cisplatin or carboplatin) +/- SRS/WBRT	Camrelizumab combined with chemotherapy (pemetrexed or paclitaxel or Nab-paclitaxel + cisplatin or carboplatin) +/- SRS/WBRT	iPFS

NA, not applicable; fr, fractions; WBRT, Whole Brain Radiation Therapy; SRS, Radiosurgery; SRT, fractionated stereotactic radiotherapy; EGFR, Epidermal Growth Factor Receptor; iPFS, Progression Free Survival; ORR, Overall Response Rate; RP2D, Recommended Phase 2 Dose; IT, Immunotherapy; RN, Radionecrosis.

**Conclusions:** The role of the RT-IT combination is still unclear. The current trend favors focal treatments such as SRS alone, or adjuvant SRS over WBRT, so further investigation of this combination is still required.

## Prophylactic Cranial Irradiation (PCI)

Prophylactic cranial irradiation (PCI) is performed with the aim of preventing the occurrence of BMs in BMs-naïve patients. Its role is well-established in patients with small cell lung carcinoma (SCLC), with a demonstrated improvement in OS (149), while it is not clearly supported by current literature data for the treatment of stage III NSCLC.

The first trial dates back to 50 years ago. The VALG study identified a potential benefit of PCI in patients with NSCLC (150) with a reduced incidence of BMs by approximately 6%, but with no impact on OS. The most recent randomized phase III studies showed similar results with a significant reduction in the incidence of BMs, but no benefit on OS (151–153), except for Li’s trial (but the study did not complete the recruitment), which showed a marginal and not statistically significant benefit in median OS (31 vs 27.4 p=0.13). Three meta-analyses of randomized studies have been published in recent years and showed identical results, with a decrease in the incidence of metastasis with PCI and no difference in OS, QoL or toxicity (154).

The results of the phase 3 NVALT/DLCRG- 02 trial were recently published. They confirmed a statistically significant reduction in BM incidence (27% vs 7% at 2 years after therapy, p=0.001) and a prolonged time to BM onset (p=0.012) in favor of PCI, but again no statistically significant difference in QoL and OS (155). On the other hand, patients receiving PCI had a significant increase of cognitive disorders (19% vs 3%) and of memory impairment (G1-2 in 30% vs 8%).

In conclusion, the addition of PCI significantly prolongs PFS and BM free survival in NSCLC patients, but has no impact on OS (156). Given the potential neurotoxicity of this approach, observation is the preferred strategy in the clinical routine, with the possibility to offer salvage RT, even as focal SRS or WBRT, at the time of progression.

**Conclusions:** The actual evidence do not support PCI as a standard treatment in patients with NSCLC.

## Discussion

The management of NSCLC patients with BMs is rapidly evolving. The advent of technological advances in imaging, radiotherapy planning and delivery techniques are rapidly replacing standard WBRT with SRS or with more conformal and less toxic solution of “extended brain irradiation” in order to reduce the therapeutic burden and to improve the risk benefit ratio in patients with metastatic brain disease. This review presents the different clinical settings of CNS metastatic involvement in NSCLC patients, with a main focus on the actual evidence-based indications and on the new technological frontiers to offer a high-quality and up-to-date RT treatment. In particular, we described the multiple technical solution to offer a focal RT treatment and to prevent the neurocognitive impairment related to WBRT, when and if the literature data do not support anymore the extended irradiation of the whole brain as an upfront management of BMs. In fact, WBRT is no longer the standard treatment for many patients with BMs from NSCLC. In reason of the growing evidence and understanding on the negative effects of WBRT on neurocognitive function and QoL, alternative RT options such as SRS and SRT are increasingly considered. The main aim of this strategy is to manage BMs with a minimal therapeutic burden to delay as much as possible WBRT and all its related side effects. This approach is certainly feasible and supported by literature data in patients with 1 or few (2–4) BMs, with the possibility even to repeat multiple sessions at different times without compromising patients’ outcome. The ambitious attempt to extend the indication to SRS and SRT to patients with a poly-metastatic CNS involvement (>5 BMs) is currently under investigation. When WBRT is eventually indicated, the evolving landscape offers the opportunity to revitalize this controversial RT solution through the implementation of hippocampal sparing (eventually combined with a neuroprotector like memantine), that may significantly reduce the related risk of neurotoxicity. Moreover, the advent of target therapies and immunotherapies, whose optimal role has been well established in selected patients, offers a novel and effective tool to treat NSCLC patients with BMs. A new era in upon us and the timing of SRS, SRT, WBRT and systemic agents will need to be reassessed and refined in order to find the best combination of these approaches for each single patient on a case-by-case selection. In the era of precision medicine, it is fundamental to guide treatment selection with the available prognostic scores (like lung-mol GPA), which include clinical, molecular and cancer-related parameters in order to stratify patients’ risk and to accurately estimate their prognosis. Finally, a multidisciplinary discussion is mandatory to accurately weigh each single risk factor and to tailor the therapeutic offer to the patients’ need.

## Author Contributions

Study conception: CM and ML. Literature research: AG and MC. Manuscript preparation: CM, AG, MC and ML. Figure and table design: AG and MC. Manuscript editing and revision: all the authors. All authors contributed to the article and approved the submitted version.

## Funding

The publication fee was supported by a grant from the Department of Oncology of University of Torino (Italy).

## Conflict of Interest

The authors declare that the research was conducted in the absence of any commercial or financial relationships that could be construed as a potential conflict of interest.

## Publisher’s Note

All claims expressed in this article are solely those of the authors and do not necessarily represent those of their affiliated organizations, or those of the publisher, the editors and the reviewers. Any product that may be evaluated in this article, or claim that may be made by its manufacturer, is not guaranteed or endorsed by the publisher.
